# Associated congenital heart disease with Hirschsprung's disease: a retrospective cohort study on 2,174 children

**DOI:** 10.3389/fcvm.2023.1215473

**Published:** 2023-08-11

**Authors:** Yujian Wu, Yun Zhu, Xu Zhang, Jinqing Feng, Huimin Xia, Yan Zhang, Jia Li

**Affiliations:** ^1^ Department of Pediatric Cardiology, Guangzhou Women and Children’s Medical Center, Guangzhou Medical University, Guangzhou, China; ^2^ Guangdong Provincial Key Laboratory of Research in Structural Birth Defect Disease, Guangzhou Women and Children’s Medical Center, Guangzhou Medical University, Guangzhou, China; ^3^ Department of Pediatric Surgery, Guangzhou Women and Children’s Medical Center, Guangzhou Medical University, Guangzhou, China; ^4^ Clinical Physiology Laboratory, Research Institute of Pediatrics, Guangzhou Women and Children’s Medical Center, Guangzhou Medical University, Guangzhou, China

**Keywords:** Hirschsprung’s disease, congenital heart disease, chromosomal abnormality, complete atrioventricular canal, congenitally corrected transposition of the great arteries, double-outlet of right ventricle

## Abstract

**Objective:**

To examine the incidence and phenotypes of congenital heart disease (CHD) in a large cohort of patients with Hirschsprung's disease (HSCR).

**Study design:**

Retrospective data review of children with HSCR between 2003 and 2020 was conducted at the Provincial Key Laboratory for Structural Birth Defects in Guangzhou, Guangdong, China. HSCR was confirmed by pathological diagnosis. CHD was defined as a gross structural abnormality of the heart or intrathoracic great vessels that is of functional significance.

**Results:**

A total of 2,174 HSCR patients (84.7% males) were studied and 306 of them underwent echocardiography. Overall, 27 children (1.2%) had associated CHD. Among them, CHDs mostly presented as atrial and ventricular septal defects (*n* = 5 and 12 respectively) and patent ductus arteriosus (*n* = 4). Three patients (1.4‰) presented as a severe CHD including complete atrioventricular canal, congenitally corrected transposition of the great arteries and double-outlet of right ventricle. Among 14 patients carrying a chromosomal abnormality, CHD was detected in 4 infants (28.6%), all being mild forms of septal defects.

**Conclusions:**

Some new and severe types of CHD were found in patients with HSCR. Patients with syndromic features had higher incidence of CHD.

## Introduction

Hirschsprung's disease (HSCR) is one of the most common congenital digestive tract disorders in neonates with an incidence of approximately 1 in 5,000 live births (1). In HSCR patients, the absence of ganglion cells in a variable length of the distal gut, beginning at the internal sphincter and extending proximally, results in absent peristalsis in the affected bowel. The final diagnosis depends on rectal suction biopsy by identifying the absence of ganglion cells (2).

HSCR is caused by deficient cranio-caudal migration, proliferation, differentiation and colonization of neural crest cells in the hindgut (3). Neural crest cells are pluripotential and then gives rise to crucial cell types ancestors, based on their migratory pathway, terminal location, and differential abilities (4). Cardiac neural crest cells, a smaller specified subgroup of neural crest cells, significantly contribute to proper cardiovascular formation, including development of the smooth muscle, septation of the cardiac outflow tract, and patterning of the septa, valves and arterial truncus (5). The developmental deficiencies in cardiac neural crest cells are considered to cause a variety of cardiac malformations, i.e., congenital heart defect (CHD) (6).

CHD is the most common congenital structural disorder in newborns affecting approximately 1% of live births (7). It is also the leading cause of mortality from birth defects (8). There is a view that both HSCR and CHD could be regarded as neurocristopathies. Several studies have reported the high incidence of CHD, being about 1.4%–17% in HSCR patients (9–14). Most of the cases were mild or moderate forms, including atrial or ventricular septal defects, mild aortic or tricuspid valve abnormalities and coarctation of the aorta. The sample size of those studies was small (ranging from 53 to 207 patients). One report described the association of hypoplastic left heart syndrome with HSCR (13). Other than this, there has been no report, to the best of our knowledge, about the occurrence of severe types of CHD in HSCR patients.

Our institution has established the Provincial Key Laboratory for Structural Birth Defects with a large number of HSCR patients. We therefore implemented a 20-year retrospective study on the patients with HSCR aiming to assess the incidence and phenotypes of associated CHDs based upon a fairly large cohort.

## Methods

This study was approved by the Institutional Review Board of Guangzhou Women and Children's Medical Center (No. 2018052406). Informed consent was waived owing to the nature of this retrospective medical record review study. All HSCR patients admitted for further evaluation and/or surgical treatment to the Guangzhou Women and Children's Medical Center (Guangzhou, Guangdong province, China) between January 2003 and December 2020 were retrospectively reviewed. Inclusion criteria were patients with pathologically confirmed diagnosis of HSCR. Exclusion criteria were unavailable medical records for review.

Demographic and clinical data were collected from the patient's records. During the hospitalization, all patients were assessed by clinical observation, physical examination and further specialist investigations based on clinical features. Echocardiographic examination was performed when a cardiovascular abnormality was suspected based on symptoms, depressed pulse oximetry, heart murmur, family history of congenital heart disease or abnormal prenatal ultrasonic findings. Detailed echo-scan was performed through a transthoracic approach with pulsed, continuous and color-Doppler using echocardiographic systems.

In our cohort, 4 types of HSCR were identified according to the length of aganglionosis: short segment HSCR (aganglionosis extending up to the left descending colon), long segment HSCR (aganglionosis extending up to the right transverse or ascending colon), total colonic aganglionosis (aganglionosis extending to the whole colon) and total intestinal aganglionosis (aganglionosis extending over the jejunum with less than 20 cm of normo-ganglionic bowel).

CHD was defined as a gross structural abnormality of the heart or intrathoracic great vessels that is actually or potentially of functional significance (15), diagnosed with echocardiography by the pediatric cardiologists. In according with many other epidemiologic studies, the definition of CHD excluded functionless abnormalities of the great veins (such as persistent left superior cava), bicuspid aortic valves, mitral valve prolapse, Marfan syndrome, cardiomyopathies, and congenital arrhythmias without an associated structural heart lesion (such as long Q-T syndrome). Atrial septal defects within oval fossa, patent foramen ovale and patent ductus arteriosus throughout the first 14 days of life were also excluded because they are considered normal findings. The CHD patients were grouped by disease severity. The category of severe CHD included those who were severely ill in early infancy (16).

### Statistical analysis

SPSS for Windows, version 26.0 (SPSS Inc, Chicago, Illinois, USA) was used. Statistical analysis was performed using 2-tailed student *t*-test (for continuous variables) and Pearson's *χ*^2^ test or Fisher's exact test (for categorical variables). A *P*-value lower than 0.05 was considered significant.

## Results

### Demographic and clinical characteristics of the cohort

**Figure 1:**
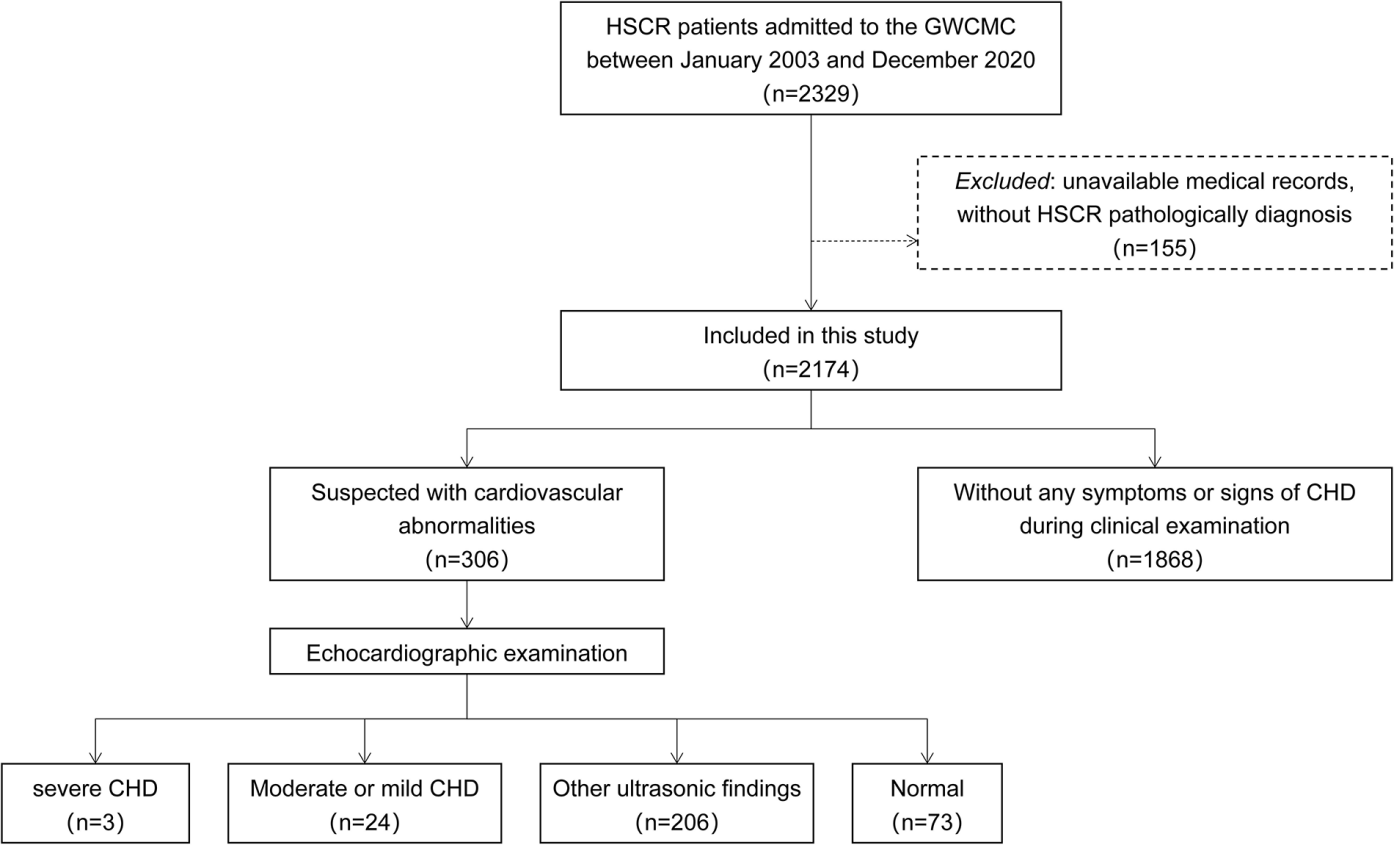
Flow chart of the study method. GWCMC, Guangzhou Women and Children's Medical Center; HSCR, Hirschsprung's disease; CHD, congenital heart disease.

A total of 2,174 patients were enrolled in this study (Figure 1). Male to female ratio was 5.5:1. Among them, 1,587 patients (72.9%) were affected by the short segment HSCR, 420 patients (19.3%) by the long segment HSCR, 129 patients (5.9%) by the total colonic aganglionosis, and 38 patients (1.7%) by the total intestinal aganglionosis. There were 306 patients undergoing transthoracic echocardiography during the study period. The lengthwise types of aganglionosis did not differ between patients with and without CHD findings (*P* = 0.051). Patients with HSCR associated with CHD had a higher proportion of syndromes compared to non-CHD patients (*P* < 0.001, Table 1).

**Table 1:** Summary of syndromic and HSCR characteristics of overall patients grouped by CHD status.

	HSCR without CHD or without echocardiography *N* = 2,147	HSCR with CHD *N* = 27	*P* value
Syndrome	10 (0.5%)	4 (14.8%)	<0.001
Type of HSCR			0.051
S-HSCR	1,572 (73.2%)	15 (55.6%)	
L-HSCR	412 (19.2%)	8 (29.6%)	
TCA	127 (5.9%)	2 (7.4%)	
TIA	36 (1.7%)	2 (7.4%)	

HSCR, Hirschsprung's disease; CHD, congenital heart disease; S-HSCR, short segment HSCR; L-HSCR, long segment HSCR; TCA, total colonic aganglionosis; TIA, total intestinal aganglionosis.

There was no significant difference in sex or birthweight between individuals with and without CHD (*P* = 0.058 and 0.432 respectively, Table 2). The gestational age in patients with CHD was smaller (37.95 ± 2.07 vs. 38.86 ± 1.75, *P* = 0.041, Table 2).

**Table 2:** Birth characteristics of overall HSCR patients with echocardiography.

	HSCR without CHD *N* = 279	HSCR with CHD *N* = 27	*P*-value
Male	236 (84.6%)	19 (70.4%)	0.058
Birth weight (kg)	3.18 ± 0.46	3.11 ± 0.63	0.432
Gestational age (w)	38.86 ± 1.75	37.95 ± 2.07	0.041

HSCR, Hirschsprung's disease; CHD, congenital heart disease.

### Details of HSCR with associated CHD and chromosomal abnormality

The overall concomitant CHDs were detected in 1.2% (*N* = 27) of the HSCR patients (Table 3 for details). Echocardiography in the 306 patients showed that CHDs were mostly presented as septal defects, i.e., 12 patients had ventricular septal defect and 5 patients had atrial septal defect. Patent ductus arteriosus was detected in 4 patients, coarctation of the aorta in 2 and pulmonary stenosis in 1. Three patients (1.4‰) had a severe CHD, including complete atrioventricular canal, congenitally corrected transposition of the great arteries and double-outlet of right ventricle. Fourteen patients had chromosomal abnormalities, including Down's syndrome (*N* = 7), Waardenburg syndrome (*N* = 3), Cri-du-chat syndrome (*N* = 2), congenital central hypoventilation syndrome (*N* = 1) and Mowat–Wilson Syndrome (*N* = 1). Among these patients, CHD was detected in 4 patients (28.6%) being atrial (*N* = 1) and ventricular septal defects (*N* = 3). Out of 7 patients with Down's syndrome, 3 (42.9%) had atrial (*N* = 1) or ventricular septal defect (*N* = 2).

**Table 3:** Details of overall HSCR patients.

CHD diagnosis	No. of patients	Type of HSCR (*N*)	Syndrome (*N*)
Severe CHD (*N* = 3)			
CAVC	1	S-HSCR (1)	
C-TGA	1	L-HSCR (1)	
DORV	1	S-HSCR (1)	
Moderate or mild CHD (*N* = 24)			
Large VSD	4	S-HSCR (2), L-HSCR (2)	Down (1)
Small VSD	8	S-HSCR (4), L-HSCR (2), TCA (1), TIA (1)	Down (1), Cri-du-chat (1)
ASD	5	S-HSCR (4), L-HSCR (1)	Down (1)
PDA	4	S-HSCR (1), L-HSCR (2), TCA (1)	
COA	2	S-HSCR (2)	
PS	1	TIA (1)	
Other ultrasonic findings (*N* = 206)			
PFO or ASD within oval fossa	191	S-HSCR (123), L-HSCR (40), TCA (20), TIA (8)	Down (1), Cri-du-chat (1)
PDA < 14 days	15	S-HSCR (9), L-HSCR (4), TCA (2)	
Normal (*N* = 73)		S-HSCR (51), L-HSCR (13), TCA (3), TIA (6)	Waardenburg (2)
Without echocardiography (*N* = 1,868)		S-HSCR (1,389), L-HSCR (355), TCA (102), TIA (22)	Down (3), Waardenburg (1), CCHS (1), MWS (1)

HSCR, Hirschsprung's disease; CHD, congenital heart disease; CAVC, complete atrioventricular canal; C-TGA, congenitally corrected transposition of the great arteries; DORV, double-outlet of right ventricle; VSD, ventricular septal defect; ASD, atrial septal defect; PDA, patent ductus arteriosus; COA, coarctation of the aorta; PS, pulmonary stenosis; PFO, patent foramen ovale; S-HSCR, short segment HSCR; L-HSCR, long segment HSCR; TCA, total colonic aganglionosis; TIA, total intestinal aganglionosis; CCHS, congenital central hypoventilation syndrome; MWS, Mowat-Wilson Syndrome.

## Discussion

HSCR was first described in 1,886 by a Danish physician, Harald Hirschsprung, and the chief culprit was revealed as aganglionosis in all or distal part of the colon. Colonic lesions fail to relax, causing functional colonic obstruction over time (17). HSCR occurs as an isolated phenotype in most cases, while a number of HSCR-associated anomalies such as gastrointestinal, neurologic, and genitourinary anomalies have been reported (10). In particular, concomitant CHDs have been drawn attention on account of its influence on the long-term prognosis.

Several studies over the last few decades have reported CHD in fairly small populations of HSCR. A prospective observational study published in 2013 detected a prevalence rate of 4.7% out of 106 children with HSCR, with coarctation of the aorta in 1 and septal defects in 4 cases (9). In another prospective study, 133 consecutive HSCR patients underwent cardiac screening and 11 patients (8.3%) presented CHDs which were mostly septal defects or small patent ductus arteriosus (14). Two epidemiological studies reported the incidence of 4/207 (1.4%, 3 with ventricular septal defect and 1 with patent ductus arteriosus) and 2/126 (1.6%, no details given) respectively (10, 18). One report showed higher incidence of HSCR in patients with hypoplastic left heart syndrome (13). In our largest cohort of patients with HSCR ever reported, we found that 1.2% of children had concomitant CHD, which were mostly presented by atrial or ventricular septal defects. The incidence of severe CHD was 1.4‰ (*N* = 3), including complete atrioventricular canal, congenitally corrected transposition of the great arteries and double-outlet of right ventricle. None of them has ever been reported. In patients with syndromic disorders, the prevalence of associated CHD was 28.6%. Among 7 patients with Down's syndrome, 42.9% had associated CHD, in concordance with previous studies (9, 14, 19).

There are several reasons which could lead to a wide range in estimates of incidence. It may be attributed to the dissimilarities in definition of CHD and methods among the studies (16). High incidences were found in the prospective studies, as they detected mild or moderate CHDs, which are often asymptomatic and do not have significant murmurs. By contrast, estimates of prevalence of severe CHD are more reliable, since that few patients in the severe group will be misdiagnosed in the children's medical centers. Most epidemiological studies reported an estimated number of 1.0–1.7 cases per 1,000 live births for severe CHD (16, 20, 21). A similar incidence was found in our HSCR population.

More interestingly, 2 of the severe CHDs were conotruncal developmental anomalies, i.e., congenitally corrected transposition of the great arteries and double-outlet of right ventricle. This may be expected as the critical embryologic role of neural crest cells in the development of both enteric nervous system and cardiac outflow tract and septation. After undergoing epithelial-mesenchymal transition, multipotent cells of the neural crest migrate and subsequently differentiate into a wide variety of cell type ancestors that are responsible for the development of various organs (22, 23). Various animal models have repeatedly confirmed that vagal neural crest cells are the main source of the enteric nervous system constituents (3, 24). The crucial role of neural crest cells in conotruncal separation during proper heart formation have been demonstrated (5, 6, 25). Although progress has been made in revealing possible embryological connection between CHD and HSCR, the developmental processes of neural crest cells are extraordinary complexities. Future experimental work in this area is needed to determine the regulatory pathways and factors during the development of cardiovascular and enteric nervous system.

In our cohort, 14 patients (0.6%) had Down's syndrome or other syndromes. Several particular reasons probably account for the lower than reported percentage (4.3%–6.6%) of Down's syndrome in our live-born series (9, 10, 14, 19). In China, prenatal screening for Down's syndrome is offered as part of routine antenatal care, and termination of pregnancy for Down's syndrome is legal (26, 27). From the viewpoint of Down's syndrome, the most frequent associated anomalies are cardiac disorders, which has been reported to occur in roughly 44%–60% (28, 29). Besides, the clinical association between Down's syndrome and HSCR has been well-established (30, 31). However, the general incidence of CHD associated with HSCR-Down's syndrome has not substantially changed compared with all Down's syndrome patients – 3 out of 7 HSCR-Down's syndrome patients (42.9%) presented also an associated CHD and similar proportions in other studies (36.1%–62.5%) (9, 14, 19). The past three nationwide surveys in Japan found that cardiovascular anomalies were increased over time among HSCR-Down's syndrome patients (19). The incremental trends were consistent with the increasing diagnosed incidence in general population over the years, which primarily due to the rising frequency of echocardiographic ultrasound screening and the growing ability to detect mild lesions. Nonetheless, the associations between CHD, HSCR and genetic syndromes are still of significance in revealing the possible abnormal genetic background and pathogenesis and in evaluating the prognosis.

Our study has several limitations. The observational retrospective study had intrinsic risks of information bias and temporal bias, and the specific biological mechanisms are speculative. Besides, our echocardiographic evaluation was conducted in only 306 patients depending on referral, which tended to underestimate the number of mild lesions, particular atrial septal defect. But it is unlikely that major cardiac defects were missed. In addition, it represents a single-institutional experience for a referral center for HSCR and should extrapolate with care. A meta-analysis combining numerous studies may help to identify further associations. Furthermore, missing information about long-term follow-up resulted in inability to assess the effect of associated CHDs on the outcomes of patients with HSCR.

## Conclusions

Our retrospective study during the last twenty years in China demonstrated CHDs in 1.2% of 2,174 HSCR patients. Echocardiography evaluation in 306 patients helped to identify 3 patients (1.4‰) with severe CHD including complete atrioventricular canal, congenitally corrected transposition of the great arteries and double-outlet of right ventricle, which have never been reported.

## Data Availability

The original contributions presented in the study are included in the article/Supplementary Materials, further inquiries can be directed to the corresponding authors.
